# Proteomic profiling reveals immunomodulatory role of IL-33 in ocular bacterial and fungal infections

**DOI:** 10.1128/iai.00183-25

**Published:** 2025-06-13

**Authors:** Zeeshan Ahmad, Sukhvinder Singh, Dhanwini Rudraprasad, Joveeta Joseph, Nikhlesh K. Singh, Ashok Kumar

**Affiliations:** 1 Department of Ophthalmology, Visual and Anatomical Sciences, Wayne State University School of Medicine 2954, Detroit, Michigan, USA; 2 Jhaveri Microbiology Centre, Brien Holden Eye Research Centre, L.V. Prasad Eye Institute, Hyderabad, Telangana, India; 3 Integrative Biosciences Center, Wayne State University 2954, Detroit, Michigan, USA; 4 Department of Biochemistry, Microbiology, and Immunology, Wayne State University School of Medicine 2954, Detroit, Michigan, USA; NIH, NIAID, Washington, DC, USA

**Keywords:** endophthalmitis, proteomic profiler, inflammation, cell death, retina, IL-33

## Abstract

Bacterial and fungal pathogens are major causes of infectious endophthalmitis following eye surgery or trauma, often leading to vision impairment or blindness. The distinct clinical outcomes observed in bacterial and fungal endophthalmitis suggest differences in host immune responses. To investigate these differences, we utilized cytokine arrays and murine models of bacterial (*Staphylococcus aureus*) and fungal (*Aspergillus fumigatus*) endophthalmitis. Our analysis revealed that cytokine responses peaked in bacterial infections at 12–24 h, whereas fungal infections exhibited a delayed peak at 48 h. Several inflammatory mediators, including MMP9, MMP3, CD14, LIX, LCN2, retinol-binding protein 4, ICAM1, and VCAM1, were differentially elevated. Notably, interleukin-33 (IL-33) levels peaked early in bacterial infections but continued to rise throughout all time points in fungal endophthalmitis. Analysis of patient vitreous samples further confirmed higher levels of IL-33 in bacterial (*n*=40) and fungal (*n*=20) endophthalmitis cases. Functional studies in IL-33-deficient mice revealed an increased fungal burden and elevated TNF-α and IL-6 levels, but bacterial endophthalmitis severity remains largely unaffected. Additionally, bone marrow-derived macrophages from IL-33^−/−^ mice exhibited increased cell death in response to fungal and bacterial infection. Our findings reveal divergent innate immune responses between bacterial and fungal endophthalmitis and emphasize the immunomodulatory function of IL-33 in ocular infections.

## INTRODUCTION

Endophthalmitis is a severe inflammatory condition of the intraocular tissues, predominantly caused by bacterial and fungal pathogens during ocular surgeries or trauma to the eye (1, 2). This sight-threatening emergency can rapidly progress, leading to substantial vision impairment or permanent blindness if not diagnosed and treated effectively (3–5). Although bacterial and fungal endophthalmitis share overlapping clinical symptoms, they elicit significantly different immune responses (6, 7). Bacterial infections prompt a rapid neutrophil response, whereas fungal infections elicit a slower, macrophage-driven reaction, affecting disease progression and therapeutic outcomes (8, 9). Current treatment options, which primarily involve broad-spectrum antibiotics or antifungals, do not fully address the nuances of pathogen-specific immune responses due to differences in immune evasion mechanisms employed by bacterial and fungal pathogens, such as biofilm formation and modulation of innate immune response (10, 11). This gap highlights the critical need for an in-depth comparative analysis of immune responses in bacterial versus fungal endophthalmitis, aimed at developing more precisely targeted and pathogen-specific therapeutic strategies (12–14).

Research from our laboratory and others underscores the pivotal role of immune dynamics in the pathogenesis and resolution of endophthalmitis (15, 16). This immune response involves a coordinated interplay between innate and adaptive mechanisms, which aim to eliminate pathogens while preserving retinal tissue integrity (17, 18). Key players include cytokines, chemokines, and immune cells that collectively orchestrate the inflammatory environment and drive host defense (19–21). Bacterial and fungal endophthalmitis, though both inflammatory, elicit distinct immune responses due to key structural differences, such as peptidoglycan in bacterial cell walls versus chitin in fungal cell walls (17, 22). To evade the immune system, bacterial and fungal pathogens employ unique structural strategies: bacterial peptidoglycan engages Toll-like receptors and NOD-like receptors to manipulate immune recognition, while fungal chitin activates C-type lectin receptors (CLRs) and dectin-1, each tailored to recognize and respond to fungal invaders (23–25).

Additionally, these pathogens differ in virulence factors—bacteria produce toxins that can activate inflammasomes and other pro-inflammatory pathways, while fungi form invasive hyphae that trigger phagocytic responses and can induce adaptive immunity (6, 26, 27). Moreover, microbial pathogens, including viruses, bacteria, and parasites, bind to cell surface heparan sulfate proteoglycans to promote their initial attachment and subsequent cellular entry (28, 29). Therefore, understanding these pathogen-specific entry and immune responses may provide insights into targeted therapeutic approaches for endophthalmitis.

This study aims to delineate and compare the immune responses during bacterial and fungal endophthalmitis through comprehensive profiling of cytokines by leveraging high-throughput immunological assays such as cytokine arrays (30). By mapping the distinct immunopathological landscapes induced by bacterial and fungal pathogens, we seek to identify potential biomarkers and therapeutic targets to enhance treatment specificity and efficacy (31). A deeper understanding of these immune pathways may guide the development of adjunctive therapies to modulate immune responses, minimize tissue damage, and ensure effective infection control (32). Such therapies could modulate the immune response to minimize tissue damage while ensuring effective control of infection (33). Ultimately, targeted therapeutic strategies that enhance host defenses could improve clinical outcomes in endophthalmitis and other infectious diseases

## RESULTS

### Temporal analysis of inflammatory mediators during bacterial endophthalmitis

**Fig 1:**
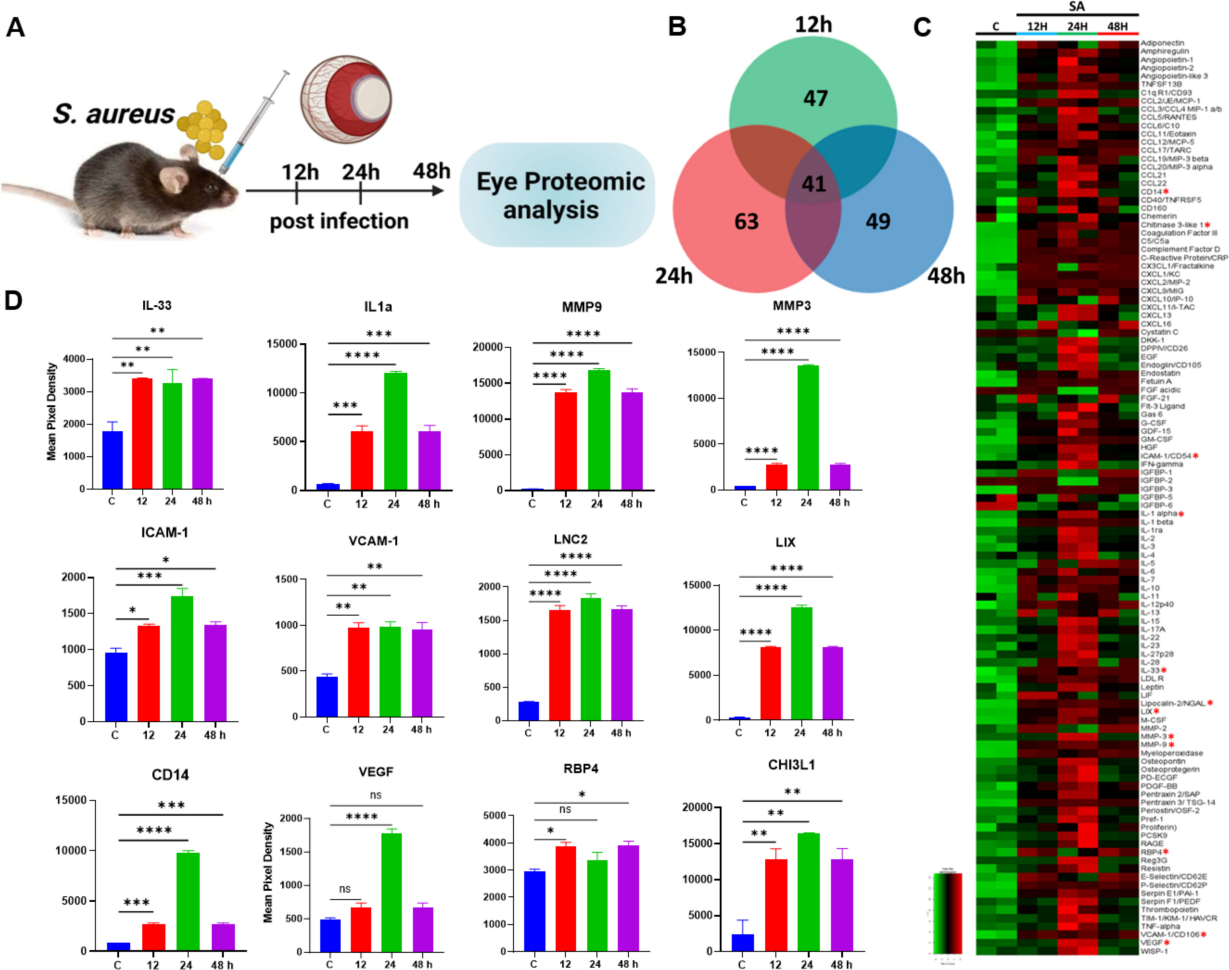
Temporal proteome profiling of whole-eye lysate during *S. aureus* endophthalmitis. Endophthalmitis was induced in the eyes (*n* = 4) of B6 mice by intravitreal inoculation of *S. aureus* (SA) RN6390 (5,000 CFUs/eye). After 12, 24, and 48 h post-infection, whole eyes were enucleated for proteome profiler analysis, and PBS-injected mice were used as a control (**C**). (**A**) Schematic representation of the experimental design. (**B**) Venn diagram illustrating the overlap and distinct sets of cytokines differentially expressed at the indicated time points. (**C**) Heatmap depicting the expression patterns of proteins across the time points. (**D**) Representative temporal profiles of key inflammatory mediators. The data represented are the culmination of two independent experiments and are shown as means ± SD. Statistical analysis was performed using one-way ANOVA with Tukey’s multiple comparison tests by comparing infected samples: (∗) *P* < 0.05; (∗∗) *P* < 0.01; (∗∗∗) *P* < 0.001; (∗∗∗∗) *P* < 0.0001.

**Fig 2:**
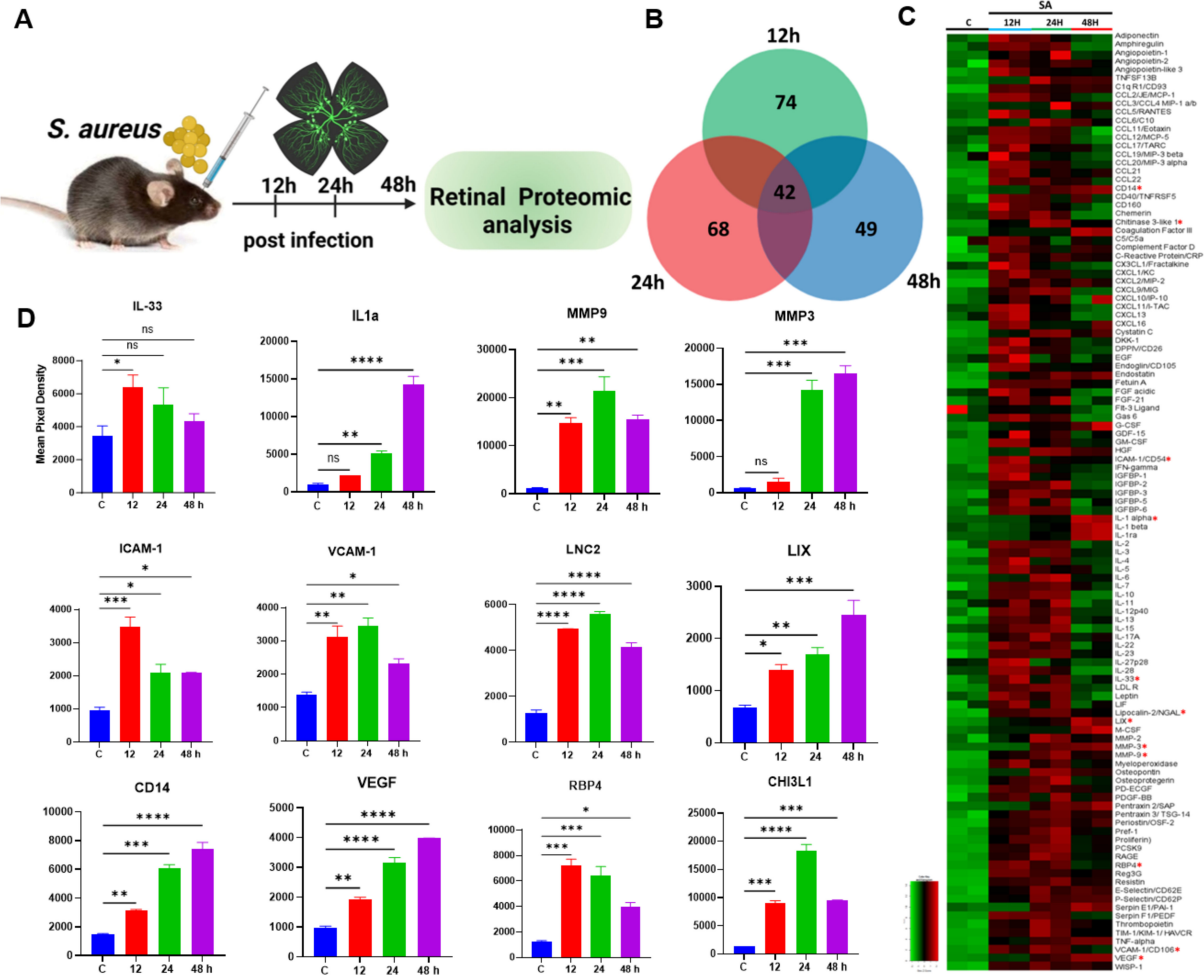
Temporal proteome profiling of retinal lysate during *S. aureus* endophthalmitis. Eyes (*n* = 4). Endophthalmitis was induced in the eyes of B6 mice by intravitreal inoculation of *S. aureus* (SA) RN6390 (5,000 CFUs/eye). After 12, 24, and 48 h post-infection, retinal tissues were harvested for proteome profiler analysis, and PBS-injected mice were used as a control (**C**). (**A**) Schematic representation of the experimental design. (**B**) Venn diagram illustrating the overlap and distinct sets of cytokines differentially expressed at the indicated time points. (**C**) Heatmap depicting the expression patterns of proteins across the time points. (**D**) Representative temporal profiles of key inflammatory mediators. The data represented are the culmination of two independent experiments and are shown as means ± SD. Statistical analysis was performed using one-way ANOVA with Tukey’s multiple comparison tests by comparing infected samples: (∗) *P* < 0.05; (∗∗) *P* < 0.01; (∗∗∗) *P* < 0.001; (∗∗∗∗) *P* < 0.0001.

We have previously reported global metabolomics (34) and lipidomic (23) perturbations, demonstrating their potential to uncover new therapeutic targets during endophthalmitis. Here, we aimed to investigate protein-level changes, particularly inflammatory mediators, during bacterial and fungal endophthalmitis. First, we performed cytokine proteome analysis in bacterial (*Staphylococcus aureus*)-infected whole-eye lysates at various time points (12, 24, and 48 h) post-infection (Fig. 1A). A Venn diagram was generated to visualize differentially produced cytokines (Fig. 1B), revealing the upregulation of 47 cytokines at 12 h, 63 at 24 h, and 49 at 48 h. The relative spot intensities of these cytokines are provided in the supplementary data (Fig. S1). These findings suggest that *S. aureus* infection elicits a robust innate immune response, peaking at 24 h post-infection. A heatmap was generated to visualize the global changes in the expression of all 111 cytokines across different time points post-infection (Fig. 1C). To gain further insight, we analyzed 41 common inflammatory mediators that were elevated at various time points and plotted 12 representative mediators. This analysis revealed significant elevations in cytokines, including members of the interleukin-1 (IL-1) family (e.g., IL-33 and IL-1α), matrix metalloproteinases (MMP9 and MMP3), adhesion molecules (ICAM1 and VCAM1), and other key proteins such as the iron-regulatory protein lipocalin-2 (LCN-2), CXCL5 (LIX), retinol-binding protein 4 (RBP4), chitinase 3-like 1 (CHI3L1), vascular endothelial growth factor (VEGF), and cluster of differentiation (CD14) (Fig. 1D). Given that our experimental model involves bacterial inoculation into the vitreous cavity, we hypothesized that inflammatory mediator levels might differ between the vitreous and retinal tissue. To investigate this, we performed temporal cytokine proteome analysis using retinal tissue lysates (Fig. 2A). The Venn diagram showed upregulation of 74, 68, and 49 cytokines at 12-, 24-, and 72 h post-infection, respectively (Fig. 2B). Heatmap analysis of all 111 cytokines indicated a marked upregulation at 12 and 24 h, followed by a decline at 48 h (Fig. 2C). Similar to whole-eye lysates, levels of all 12 representative mediators (IL-33, IL-1α, MMP9, MMP3, VCAM1, ICAM1, LCN-2, LIX, RBP4, CHI3L1, VEGF, and CD14) were elevated in infected retinal tissue (Fig. 2D). The relative spot intensities of these mediators are provided in the supplementary data (Fig. S2). Comparative analysis of whole-eye versus retinal lysates indicated relatively higher levels of inflammatory mediators in retinal tissue, except IL-1α, LIX, CD14, and CHI3L1 (Fig. S3). Collectively, our proteomic profiling revealed differential expression of inflammatory mediators with distinct temporal patterns, which may correlate with disease progression during *S. aureus* endophthalmitis.

### Temporal analysis of inflammatory mediators during fungal endophthalmitis

**Fig 3:**
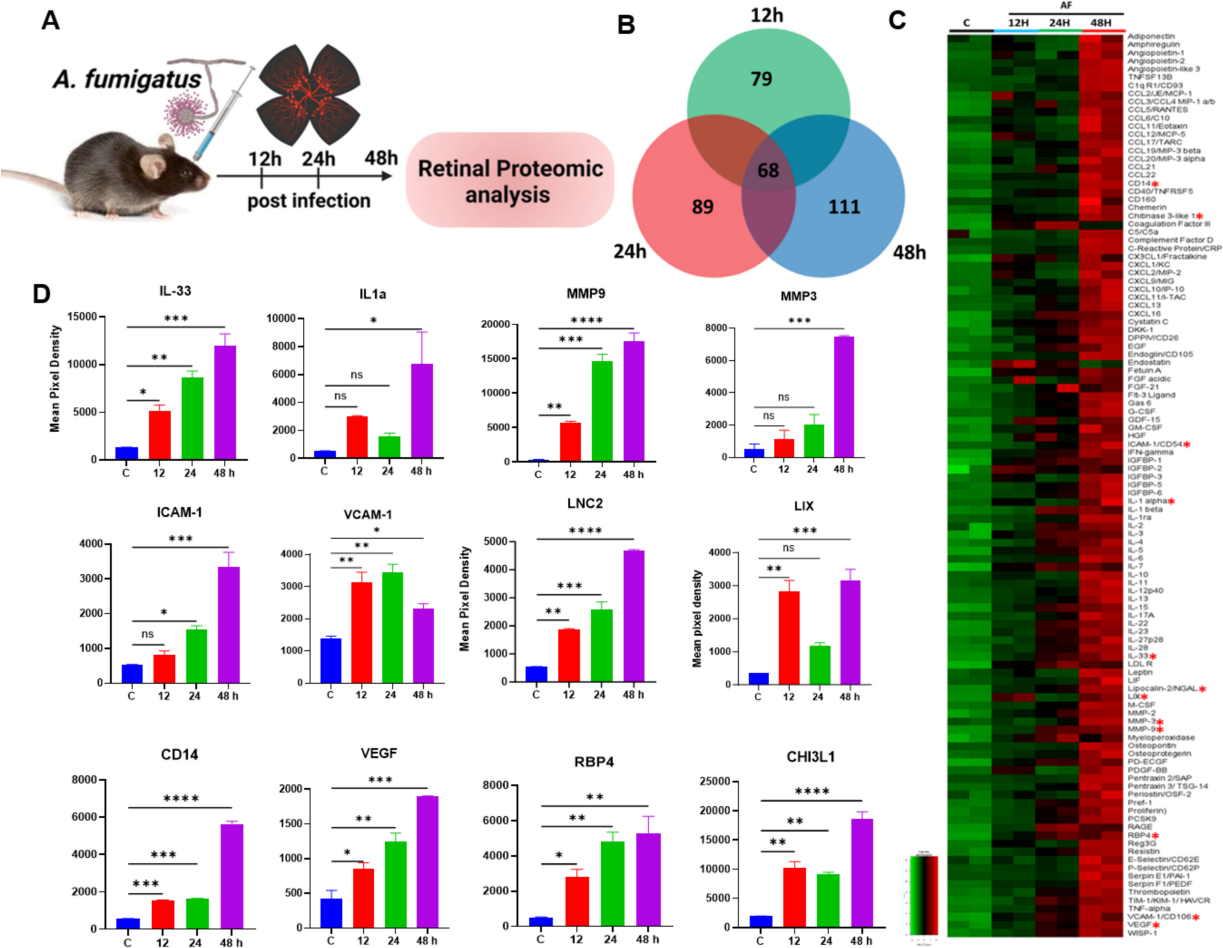
Temporal proteome profiling of retinal lysate during *A. fumigatus* endophthalmitis. Eyes (*n* = 4). Endophthalmitis was induced in the eyes of B6 mice by intravitreal inoculation of *A. fumigatus* (15,000 CFUs/eye). After 12, 24 h, and 48 h post-infection, retinal tissues were harvested for proteome profiler analysis, and PBS-injected mice were used as a control (**C**). (**A**) Schematic representation of the experimental design. (**B**) Venn diagram illustrating the overlap and distinct sets of cytokines differentially expressed at the indicated time points. (**C**) Heatmap depicting the expression patterns of proteins across the time points. (**D**) Representative temporal profiles of key inflammatory mediators. The data represented are the culmination of two independent experiments and are shown as means ± SD. Statistical analysis was performed using one-way ANOVA with Tukey’s multiple comparison tests by comparing infected samples: (∗) *P* < 0.05; (∗∗) *P* < 0.01; (∗∗∗) *P* < 0.001; (∗∗∗∗) *P* < 0.0001.

**Fig 4:**
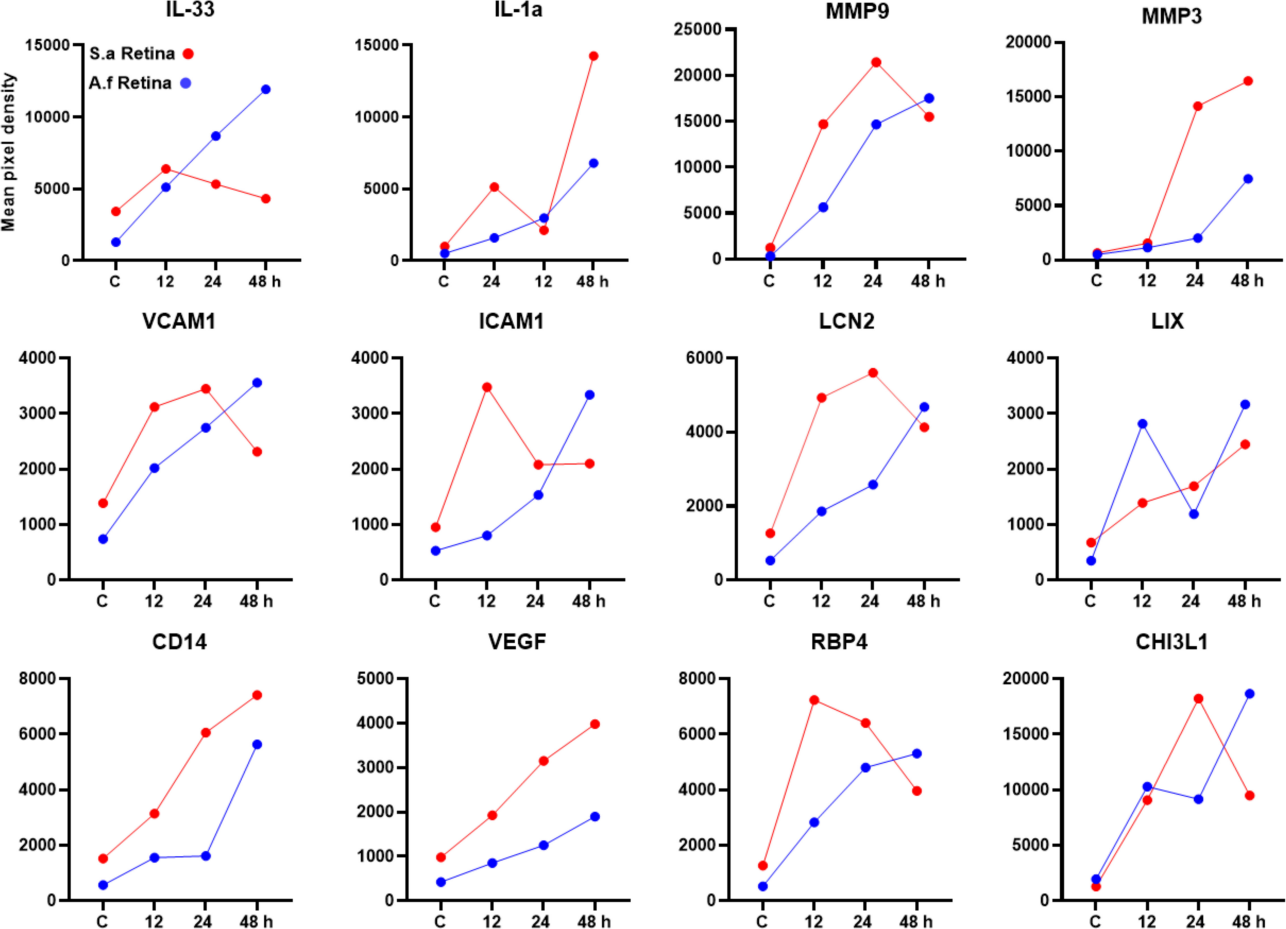
Comparative analysis of proteins in bacterial vs fungal endophthalmitis. The protein profiles of *S. aureus* (red line) and *A. fumigatus* (blue line)-infected retinal lysates at 12, 24, and 48 h post-infection were compared head-to-head to visualize the relative expression. The data represented are the culmination of two independent experiments and are shown as means.

In addition to bacterial pathogens, fungi are the second leading cause of endophthalmitis in certain geographical locations (35). Next, we sought to determine cytokine profiling during fungal endophthalmitis using our established mouse model of *Aspergillus fumigatus* endophthalmitis (12, 33), the most common cause of exogenous fungal endophthalmitis. To achieve this, we performed a proteomic profiler analysis using retinal tissue from *A. fumigatus*-infected and uninfected (mock) mouse eyes (Fig. 3A). A Venn diagram illustrated the overlap and unique expression patterns of cytokines at various stages of infection. Our analysis demonstrates that at 12 h post-infection, 79 cytokines were upregulated, and this response was further amplified with higher levels of 89 and 111 cytokines at 24 and 48 h post-infection, respectively (Fig. 3B). These observations were corroborated by the heatmap analysis showing marked upregulation of all 111 inflammatory mediators at 48 h (Fig. 3C). Among the key representative cytokines, we observed a time-dependent increase in levels of IL-33, MMP-9, MMP-3, ICAM-1, VCAM-1, LCN-2, RBP4, CHI3L1, VEGF, and CD14, whereas the levels of IL-1α and LIX fluctuated (reduced) at 24 h (Fig. 3D). Among these, 68 cytokines were consistently elevated (≥1.5-fold) across all time points (Table S1). The relative intensities of these cytokine dot-blots are presented in the supplementary (Fig. S4). Comparative analysis of the retinal cytokine profile in bacterial versus fungal endophthalmitis shows relatively higher levels for most molecules during fungal infection (Fig. 4).

### Validation of proteomic profiling during bacterial and fungal endophthalmitis in mouse and human vitreous

**Fig 5:**
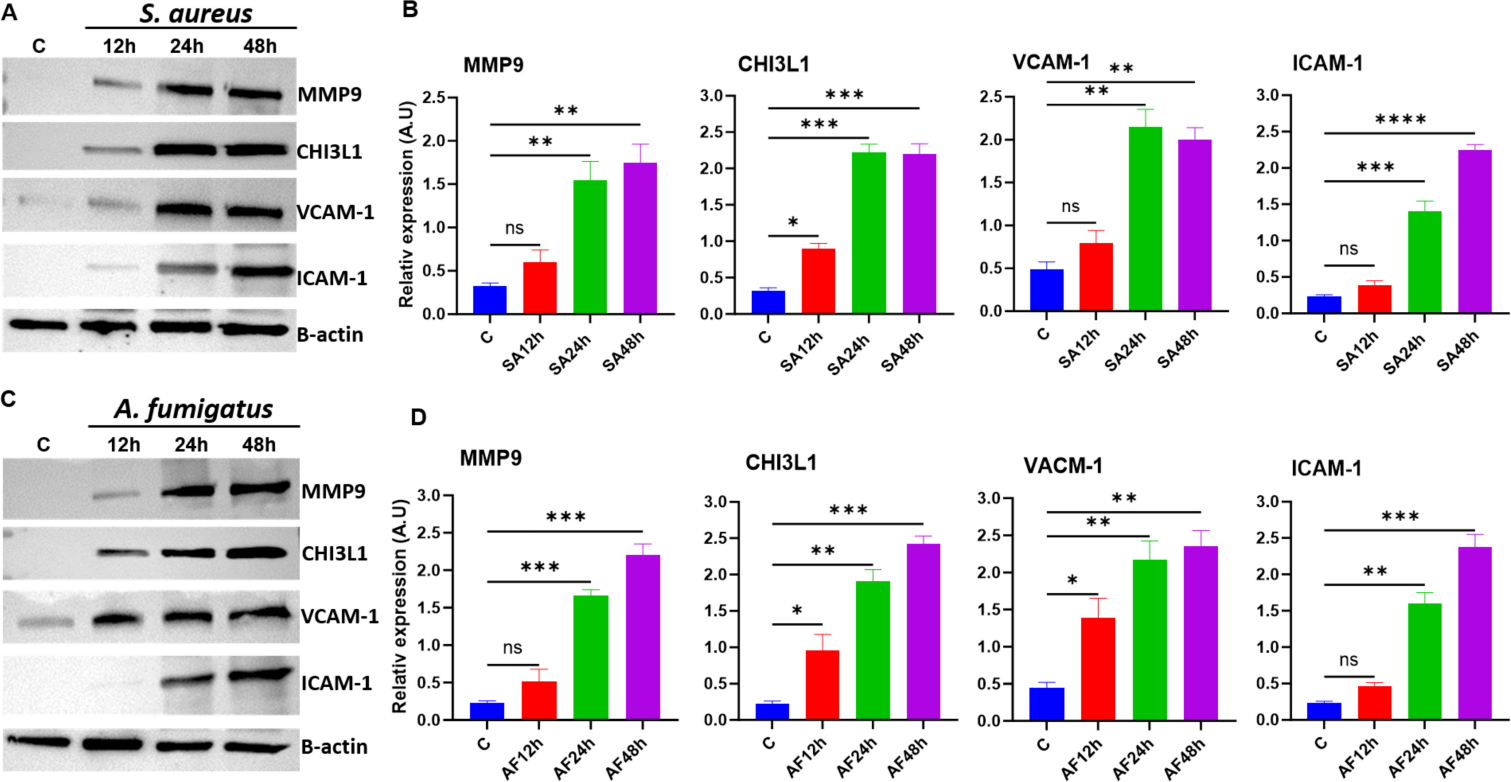
Validation of protein profiles during experimental bacterial and fungal endophthalmitis. Eyes (*n* = 4) of C57BL/6 mice were intravitreally injected with *S. aureus* (5,000 CFUs/eye) or *A. fumigatus* (15,000 CFUs/eye). Retinas were harvested at indicated time points post-infection, lysed with sonication in RIPA buffer with protease inhibitor, and subjected to western blot analysis using β-actin as the endogenous control. Western blot detection of MMP-9, CHI3L1, VCAM-1, and ICAM-1 during *S. aureus* (**A**) and *A. fumigatus* (**C**) infections. Densitometric analysis (B and D) was performed using ImageJ software, and the results are expressed as relative fold changes normalized to the respective loading control, β-actin. Statistical analysis was performed using one-way ANOVA with Tukey’s multiple comparison tests by comparing infected samples: ns, not significant; (∗) *P* < 0.05; (∗∗) *P* < 0.01; (∗∗∗) *P* < 0.001; (∗∗∗∗) *P* < 0.0001.

Our protein profiler analysis shows elevated levels of several inflammatory mediators. Consistent with the proteomic analysis, western blot showed a time-dependent increase in levels of MMP9, CHI3L1, VCAM1, and ICAM1 in *S. aureus-* (Fig. 5A and B) and *A. fumigatus* (Fig. 5C and D)-infected mouse retinal tissue, further corroborating the proteomic profiling data.

**Fig 6:**
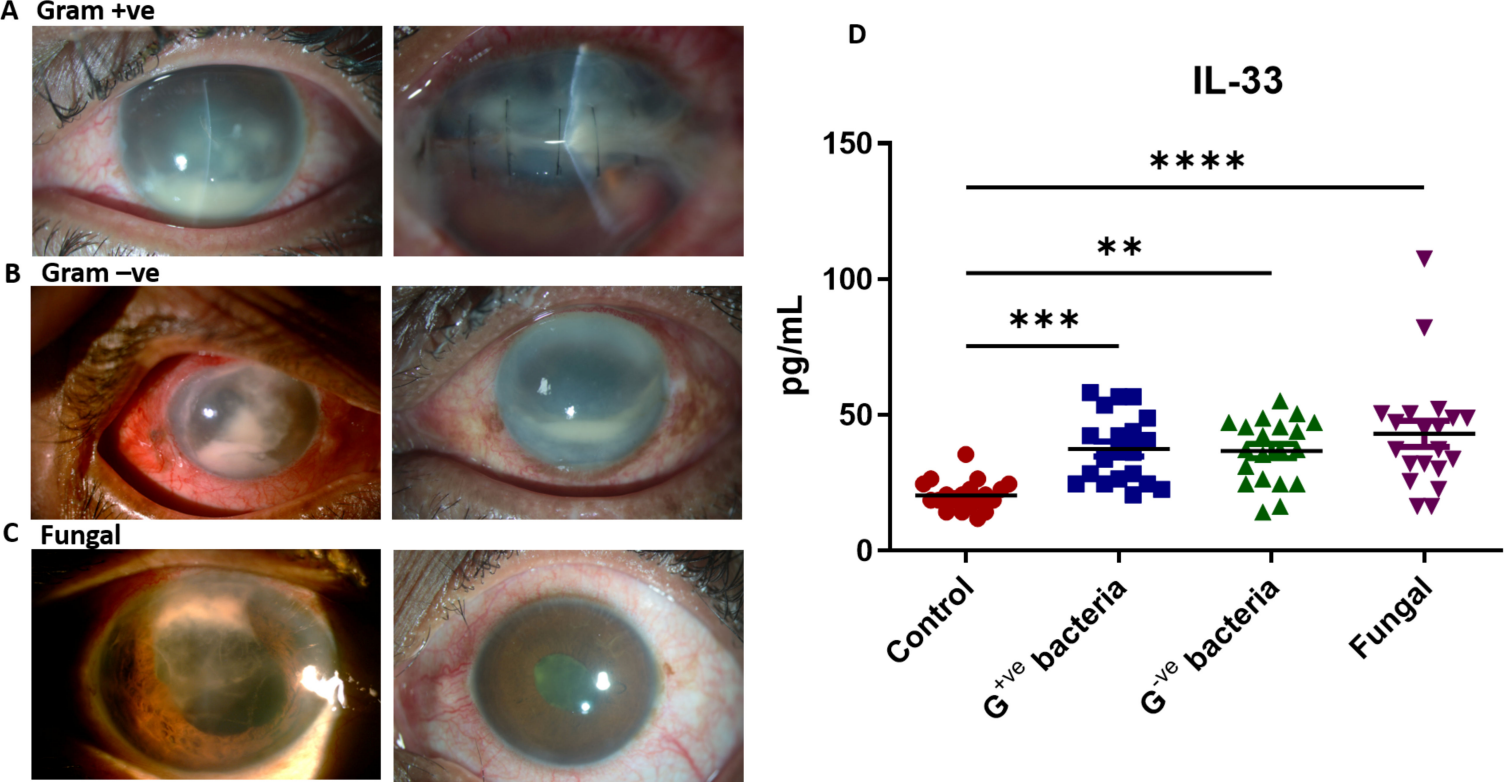
Assessment of IL-33 levels in patients with bacterial and fungal endophthalmitis. (**A–C**) Representative clinical images from patients with endophthalmitis with confirmed bacterial (gram-positive and gram-negative) or fungal endophthalmitis. (**D**) Vitreous samples (25 µL) from patients with culture-positive bacterial (*n* = 40, [gram positive and gram negative, 20 each]) and fungal (*n* = 20) endophthalmitis, along with HCs (*n* = 20), were used for the detection of IL-33 using ELISA. Statistical analysis was performed using the one-way ANOVA by comparing control with endophthalmitis samples: (∗∗) *P* < 0.01; (∗∗∗) *P* < 0.001; (∗∗∗∗) *P* < 0.0001

Given the emerging role of IL-33 in immunomodulation (36), particularly in eye diseases (37–39), and its consistent upregulation in experimental bacterial and fungal endophthalmitis, we sought to explore its yet undefined role in endophthalmitis pathogenesis. First, we assessed IL-33 production in human endophthalmitis by analyzing vitreous samples from patients with confirmed bacterial and fungal etiology. Our study included a total of 80 patients diagnosed with gram-positive (*n* = 20) and gram-negative (*n* = 20) bacterial endophthalmitis and fungal endophthalmitis (*n* = 20). Vitreous samples from 20 additional patients undergoing vitrectomy for noninfectious retinal conditions (e.g., retinal detachment, macular hole, and epiretinal membrane removal) were included as healthy controls (HCs). The average age of the gram-positive endophthalmitis cohort is 30.7 ± 23.9 years (range, 2–72 years) with 66.7% (*n* = 14) being male and 33.3% (*n* = 7) female. Twenty patients with gram-negative endophthalmitis cohort had a mean age of 45.3 ± 23.8 years (range, 4–74 years). Males accounted for 70.0% (*n* = 14) of cases, while females made up 30.0% (*n* = 6). Similarly, 20 patients with fungal endophthalmitis were analyzed, with a mean age of 44.8 ± 18.5 years (range, 2 months–68 years). The control group comprised 20 patients with a mean age of 54.4 ± 14.8 years (range, 19–72 years), with males representing 75.0% (*n* = 15) and females 25.0% (*n* = 5). A detailed demographic of the study group is provided in Table S2. The representative images of patient’s eye showed pronounced inflammatory responses, including haze, hypopyon, and corneal opacity, likely due to their rapid onset of disease progression in bacteria (Fig. 6A and B) versus fungal endophthalmitis (Fig. 6C). The enzyme-linked immunosorbent assay (ELISA) showed minimal to undetectable levels of IL-33 in HC vitreous samples. In contrast, their levels were significantly elevated in patients with fungal endophthalmitis (48.8 ± 10.2 pg/mL), followed by those with gram-positive (33.9 ± 12.5 pg/mL) and gram-negative bacterial (29.3 ± 11.7 pg/mL) endophthalmitis (Fig. 6D). These findings suggest the potential role of IL-33 in the pathogenesis of infectious endophthalmitis.

### Immunomodulatory role of IL-33 in the pathogenesis of endophthalmitis

**Fig 7:**
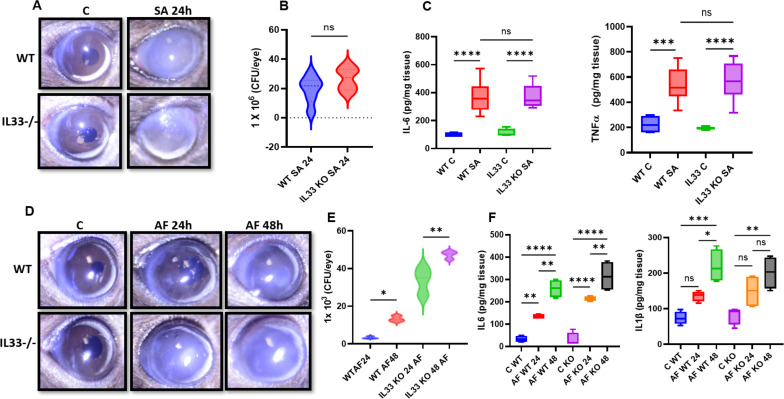
Effect of IL-33 deficiency on the pathogenesis of bacterial and fungal endophthalmitis. Endophthalmitis was induced in B6 WT and IL-33**^−/−^** mice (B6 background) by intravitreal inoculation of *S. aureus* (SA) or *A. fumigatus* (AF) (*n* = 4 per condition). (A and D) Representative slit-lamp micrograph showing corneal haze/opacity at the indicated time point post-infection. Quantitation of intraocular bacterial (**B**) and fungal (**E**) burden in whole-eye lysates by serial dilution and plate counting method. (C and F) ELISA of indicated inflammatory cytokines. The data represented are the culmination of two independent experiments and are shown as means ± SD. Statistical analysis was performed using one-way ANOVA with Tukey’s multiple comparison tests by comparing infected samples: (∗) *P* < 0.05; (∗∗) *P* < 0.01; (∗∗∗) *P* < 0.001; (∗∗∗∗) *P* < 0.0001.

Building on our findings in human endophthalmitis and experimental models, we next sought to investigate the functional role of IL-33 in the pathogenesis of infectious endophthalmitis. To achieve this, we utilized IL-33 knockout (KO) mice to assess how the IL-33 deficiency influences disease progression, inflammatory responses, and overall outcomes in bacterial and fungal endophthalmitis. In the case of bacterial endophthalmitis, our data showed that at 24 h post-S. *aureus* infection, IL-33 KO mice displayed similar disease severity to wild-type (WT) mice, indicated by comparable corneal haze, opacity, and hypopyon formation (Fig. 7A). Similarly, no significant differences were observed in bacterial burden (Fig. 7B) and levels of inflammatory mediators (TNF-α and IL-6) in KO versus WT mice (Fig. 7C). In contrast, *A. fumigatus*-infected eyes exhibited increased disease severity, i.e., corneal haze, opacity, and hypopyon in IL-33 KO mice both at 24 and 48 h post-infection (Fig. 7D). This coincided with the increased fungal burden (Fig. 7E) and elevated levels of TNF-α and IL-1β in IL-33 KO mice (Fig. 7F), indicating a protective and anti-inflammatory role of IL-33 during fungal endophthalmitis.

**Fig 8:**
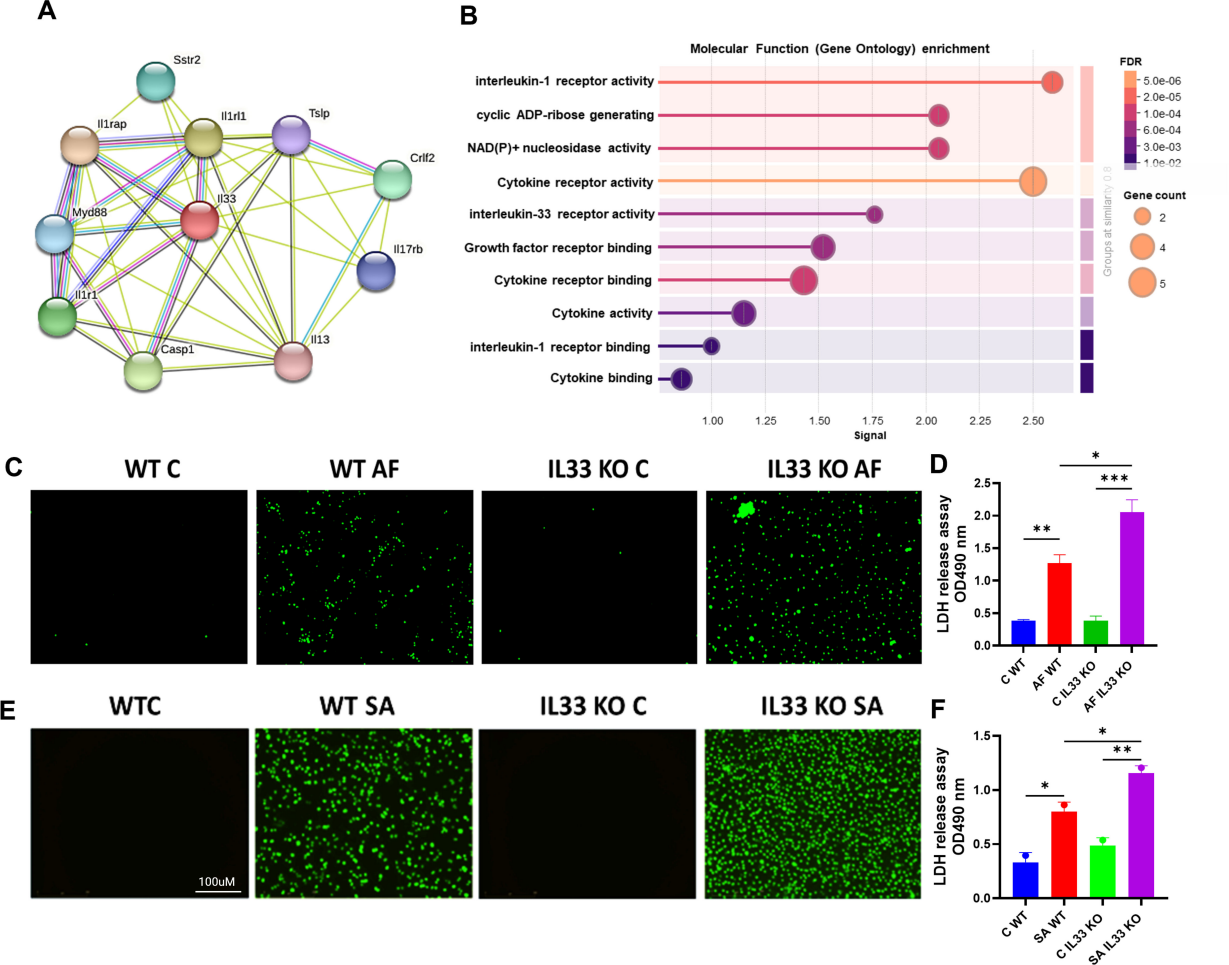
String analysis and the role of IL-33 on cell survival during fungal infection. (**A**) PPI network of IL-33 was constructed using the STRING database. (**B**) GO enrichment analysis of the STRING-derived PPI network, demonstrating IL-33’s role in regulating cell survival. Real-time analysis of cell death in BMDMs was performed using SYTOX Green dye and the Incucyte SX5 live-cell imaging system following infection with *A. fumigatus* (**C**) and *S. aureus* (**E**). LDH release was estimated from infected BMDMs following *A. fumigatus* (**D**) and *S. aureus* (**F**) infection. Data are represented as mean ± SD. Statistical analysis was performed using one-way ANOVA: (∗) *P* < 0.05; (∗∗) *P* < 0.01; (∗∗∗) *P* < 0.001; (∗∗∗∗) *P* < 0.0001.

Innate immune cells such as neutrophils and macrophages play a key role in the pathogenesis of endophthalmitis (12, 26). To better understand IL-33-associated signaling pathways, we constructed a protein-protein interaction (PPI) network for murine IL-33 using the STRING database. This analysis shows strong interactions between IL-33, Caspase-1, ILr1, MYD88, and IL1rap, indicating the involvement of IL-33 in regulating inflammasome-driven pyroptotic cell death (Fig. 8A). The gene ontology (GO) enrichment analysis of the PPI enhancement of cytokine receptor activity includes IL-1 receptor and IL-33 receptor activation (Fig. 8B). To investigate the role of IL-33 on cell death, WT and IL-33-deficient bone marrow-derived macrophages (BMDMs) were challenged with *A. fumigatus* or *S. aureus*, followed by SYTOX Green and lactate dehydrogenase (LDH) assays. The SYTOX Green assay showed an increased influx of green dye in IL-33 KO vs. WT BMDMs, indicating the loss of cellular integrity in infected cells (Fig. 8C and E). Similarly, LDH release was higher in IL-33 KO cells (Fig. 8D and F). These results indicate the cytoprotective role of IL-33 during infection.

## DISCUSSION

Both bacterial and fungal pathogens remain the leading causes of endophthalmitis following ocular surgery or trauma to the eye. Previous studies from our lab and others have reported the activation of multiple signaling pathways and inflammatory mediators during bacterial and fungal endophthalmitis (12, 34, 40–42). However, the head-to-head comparison of innate response under these conditions has not been characterized. Our study fills this gap by utilizing cytokine proteome profiling to unveil distinct innate and inflammatory responses during bacterial and fungal endophthalmitis. Moreover, we uncovered the modulation of various growth factors, extracellular matrix (ECM), iron regulatory, and scavenging proteins, which might play a role in the pathogenesis of endophthalmitis. Furthermore, our data show elevated levels of IL-33 in the vitreous of endophthalmitis patients and demonstrate its immunomodulatory role in experimental endophthalmitis.

In our experimental endophthalmitis models, pathogens were directly inoculated into the vitreous cavity of mice, where retinal resident cells, including Müller glia (43) and microglia (41), and infiltrating innate immune cells, primarily neutrophils (26), recognize the pathogen and trigger inflammatory responses (16). This initial inflammatory response is essential to combat pathogens, but excessive inflammation must be controlled to protect retinal tissues (44, 45). Understanding the complex interactions between host and pathogen is crucial to unraveling the mechanisms that drive disease progression in ocular infections (46). Our study advances the understanding of endophthalmitis by dissecting the complex cytokine interactions underlying inflammation, revealing distinct patterns of cytokine and chemokine responses during bacterial and fungal infections (31). Previous studies from our lab showed that A. *fumigatus* infection in both neutropenic and immunocompetent C57BL/6 mice led to progressive worsening of endophthalmitis, including corneal haze, opacity, and hypopyon, as early as 2 days post-infection, highlighting the aggressive nature of fungal endophthalmitis (12). Furthermore, the inherent resistance of filamentous fungi to antifungal treatment is in part due to their cell wall structures and biofilm formation capabilities (47–49). This similarity in clinical presentation between fungal and bacterial endophthalmitis often results in misdiagnoses and delays in appropriate therapy and treatment (50). Therefore, a deeper understanding of fungal endophthalmitis pathogenesis is needed to develop therapies that overcome treatment barriers (51). Our proteomic analysis provided insights into cytokine and chemokine dynamics, revealing potential therapeutic targets for modulating intraocular immune responses (52–54).

The protein profiler analysis categorized various molecules into seven major groups: cytokines, chemokines, adipokines, growth factors, iron regulatory proteins, iron scavenging proteins, and ECM proteins. This categorization led to the identification of several molecules whose roles in endophthalmitis have not yet been elucidated. For example, our data revealed elevated levels of MMP-9 and MMP-3 in both bacterial and fungal endophthalmitis. High levels of MMP-9 degrade ECM, weaken the blood-retinal barrier, and promote retinal cell apoptosis, contributing to vascular dysfunction and vision loss (55). We also reported impaired blood-retinal barriers during endophthalmitis (56); thus, targeting MMP-9 may help preserve retinal integrity and mitigate disease. The inhibition of MMP9 has been shown to protect the retina by reducing the ECM-mediated damage in ocular pathologies (57, 58). The observed elevated levels of adhesion molecules ICAM-1 and VCAM-1 during endophthalmitis corroborate with prior studies, indicating their role in retinal inflammation (1). Another interesting molecule with consistent upregulation was LCN2, which primarily sequestrates free iron, thus limiting its availability to bacteria, and *Lcn2*^−/−^ mice exhibit an increased susceptibility to bacterial infections (59). The increased levels of LCN2 in our study indicate its protective role during endophthalmitis. Our analysis also revealed notable changes in fetuin and CHI3L1. Fetuin, a glycoprotein with anti-inflammatory properties, may modulate systemic inflammation, while CHI3L1, implicated in tissue remodeling, likely plays a role in the inflammatory process. In particular, CHI3L1 has been shown to protect the eye from fungal infections (60). Together, our data implicate the potential role of these molecules in the pathobiology of endophthalmitis, and further studies are warranted to elucidate their specific contributions and therapeutic efficacy.

Among the various inflammatory mediators analyzed, we observed a distinct pattern of IL-33 production during fungal endophthalmitis. Thus, we decided to investigate its role in endophthalmitis. Previous studies have shown that IL-33-induced M2 macrophage polarization reduces retinal inflammation and even ameliorates experimental autoimmune uveitis (61), underscoring its potential as a therapeutic target in ocular inflammatory diseases. Studies have also implicated the role of IL-33 in various ocular diseases, including glaucoma (62), age-related macular degeneration (63, 64), and diabetic retinopathy (38, 65). IL-33 is released in response to cell damage or stress, as an alarmin/danger signal (66), and it also helps resolve inflammation by promoting apoptotic cell clearance and modulating pro-resolving pathways (67–69). Additionally, IL-33-deficient mice with retinal detachment show more severe cone photoreceptor degeneration, emphasizing its protective role in retinal health (70). Unlike many other secreted cytokines, IL-33 is a nuclear cytokine that is passively released in response to cell necrosis or tissue damage, serving as an alarmin to alert the immune system to injury (71). Despite IL-33’s well-established role in modulating immune responses in inflammatory conditions, its involvement in ocular infections such as endophthalmitis remains largely unexplored.

Our study revealed increased levels of IL-33 in both experimental fungal and bacterial endophthalmitis, indicating its role in modulating intraocular immune responses (72). In *A. fumigatus* endophthalmitis, IL-33 levels continue to remain elevated at all time points, where they declined during bacterial endophthalmitis. To investigate the specific role of IL-33 in endophthalmitis, we used IL-33 KO mice, and our data showed marked differences between bacterial and fungal endophthalmitis. IL-33 deficiency exacerbated disease severity in fungal but not bacterial endophthalmitis, implicating the protective role of IL-33 in preserving retinal architecture and reducing inflammation. These *in vivo* findings were corroborated by our *in vitro* experiments, where IL-33-deficient BMDMs exhibited increased cell death upon fungal and bacterial infection. IL-33 binds to its specific receptor ST2 to exert pro-inflammatory and immunomodulatory effects (73). Further studies are needed to assess the immunomodulatory role of IL-33/ST2 signaling in ocular infections.

Building on our experimental endophthalmitis models, we extended our study to human endophthalmitis by assessing IL-33 levels in their vitreous samples . Our analysis revealed that IL-33 levels were significantly elevated in all endophthalmitis patients compared to HCs, with relatively higher levels in fungal endophthalmitis cases. Among the bacterial endophthalmitis cohort, no significant differences were observed in gram-positive versus gram-negative infections. These findings further validate our mouse model data and underscore IL-33’s potential as an inflammatory marker during endophthalmitis. One of the limitations of our study is that the observed increase in IL-33 levels appears to be largely driven by two fungal endophthalmitis patient samples, which exhibited markedly higher IL-33 concentrations compared to the rest of the cohort. This heterogeneity in IL-33 levels may reflect differences in baseline immune status, the severity of intraocular inflammation, fungal burden, or the extent of retinal tissue damage among patients. Thus, further studies with larger patient cohorts are needed to define the relationship between IL-33 levels and disease progression. Another limitation of this study is the exclusive focus on experimental models of *S. aureus* and *A. fumigatus* endophthalmitis. Although both are clinically relevant pathogens, the immune response during ocular infection can differ markedly depending on the pathogen type and its virulence. Notably, gram-negative bacteria and yeast (*Candida* species) are likely to trigger distinct inflammatory responses, including IL-33 levels. While exploring these differences falls outside the current scope, future studies should include a broader spectrum of pathogens to better elucidate IL-33’s role across various types of ocular infections.

**Fig 9:**
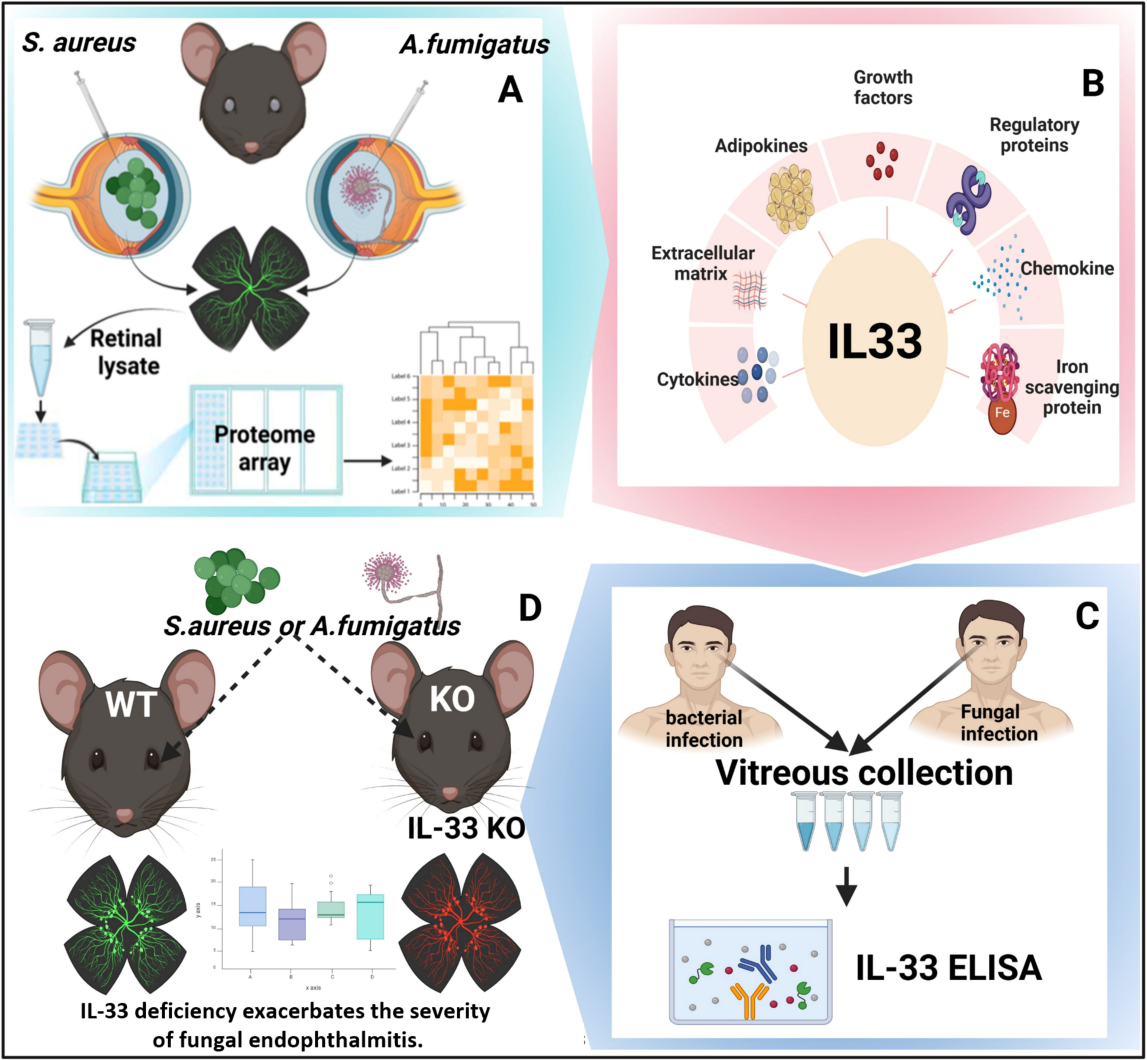
Schematic of study design. (**A**) Proteomic analysis was performed in experimental models of *S. aureus* or *A. fumigatus* endophthalmitis using whole-eye or retinal tissue lysates. (**B**) Biological and functional categorization of altered proteins, including IL-33, during endophthalmitis. (**C**) Validation of IL-33 levels in vitreous samples from bacterial or fungal endophthalmitis patients. (**D**) Functional role of IL-33 in KO mice.

In conclusion, our study offers crucial insights into the innate and inflammatory responses during bacterial and fungal endophthalmitis. Notably, we demonstrated the differential expression and protective function of IL-33 in both experimental models and human endophthalmitis patients, underscoring its potential as a newer therapeutic target (Fig. 9). Collectively, our findings enhance our understanding of endophthalmitis and pave the way for developing more effective treatment strategies to ameliorate intraocular infections.

## MATERIALS AND METHODS

### Induction of endophthalmitis

Endophthalmitis was induced in WT C57BL/6 (B6) and IL-33 KO mice via intravitreal injection of 5,000 colony forming units (CFU) of *S. aureus* (strain RN6390) or 15,000 CFU of a clinical isolate of *A. fumigatus* that was obtained from the Division of Infectious Diseases, Department of Internal Medicine, at Wayne State University School of Medicine (33). Mice were anesthetized prior to infection using a combination of ketamine (80 mg/kg) and xylazine (10 mg/kg), administered intraperitoneally, and one eye per mouse was infected as per the approved Institutional Animal Care and Use Committee (IACUC) protocol. For bacterial endophthalmitis, *S. aureus* (strain RN6390) was cultured in tryptic soy broth (TSB) at 37°C with shaking until mid-logarithmic phase (OD₆₀₀ 0.5), harvested by centrifugation, washed, and resuspended in sterile phosphate-buffered saline (PBS) for intravitreal injection. For fungal endophthalmitis, *A. fumigatus* clinical isolate was grown on Sabouraud dextrose agar (SDA) plates at 37°C for 5–7 days till sporulation. Fungal spores were harvested in sterile PBS containing 0.01% Tween 20 and enumerated using a hemocytometer and resuspended in sterile PBS for intravitreal injection. Eyes injected with the vehicle (PBS) were used as controls. At specific time points after the infection, whole eyes or retinal tissues were collected for subsequent analyses, including cytokine profiling, quantitative PCR, and western blotting.

### Bacterial and fungal burden estimation

The bacterial and fungal burden in infected eyes was performed using the serial dilution and plate count method, as reported in our prior publications (12, 33). To summarize, eyeballs were removed at specific intervals and homogenized in sterile PBS with stainless steel beads using TissueLyser II (Qiagen). The resulting mixture was diluted in sterile PBS, plated on TSB (for bacteria) or SDA (for fungi) plates, and then kept at 37°C. After overnight incubation, bacterial and fungal colonies were counted and presented as the mean CFU per eye ± standard deviation (SD).

### Isolation of bone marrow-derived macrophages

Mouse bone marrow-derived macrophages were obtained from B6 WT and IL-33 KO mice. In short, the bone marrow was collected from the tibia and femur using RPMI-1640 media with 10% fetal bovine serum (FBS) and 0.2 mM ethylenediaminetetraacetic acid (EDTA). After gentle centrifugation at 400  ×  *g* for 5 minutes at 4°C, the red blood cells were carefully removed with 0.2% NaCl solution, followed by 1.6% NaCl solution. The cells were then thoroughly rinsed and gently placed in a 100 mm tissue culture dish containing RPMI media. Macrophage differentiation was induced using 10 ng/mL macrophage colony-stimulating factor (M-CSF; Bio Legend, Cat. No. 576406) in RPMI-1640 medium supplemented with 10% FBS and 100 U/mL penicillin. Cells were cultured at 37°C in a humidified atmosphere containing 5% CO₂.

### Proteome array

The Proteome Profiler mouse XL Cytokine Array Kit (R&D Systems, MN, USA, ARY022B) was used to assess innate responses during bacterial and fungal endophthalmitis. Briefly, whole-eye or retinal tissue lysates were prepared from control and infected eyes (*n* = 4, two eyes/retina were pooled for each experiment) by sonication, and samples were processed as per the user’s manual. An equal amount (300 µg/sample in 1.5 mL) of proteins was used after Micro BCA Protein Assay Kit (Thermo Fishers Sci: Cat, 23225). The whole-eye or retinal lysates were incubated with the antibody array membranes overnight at 4°C. After washing, the membranes were incubated with a freshly prepared detection antibody cocktail. This was followed by the application of chemiluminescent reagents provided in the kit, and membranes were protected in plastic sheeting before imaging in the iBright 1500 imager (Thermo Scientific, Rockford, IL, USA). After subtracting the background from the images, the signal density of each spot was automatically calculated using the QuickSpots imaging program. The values were plotted in the graphs as the mean pixel density of each protein obtained from the two separate tests. Spot intensity analysis was performed using QuickSpots Image Analysis Software purchased from R&D Systems.

### Western blotting

The whole-eye or retinal tissue lysates were prepared using sonication followed by centrifugation at 12,000 x g for 15 minutes in PBS containing a protease and phosphatase inhibitor cocktail. Using a Micro BCA Protein Assay Kit for the total protein content of retinal lysates was measured (Thermo Scientific, Rockford, IL, USA). Total protein samples (40–50 µg) were electro-blotted onto a polyvinylidene fluoride (PVDF) membrane after being resolved on SDS-PAGE in Tris-glycine-SDS buffer (25 mM Tris, 250 mM glycine, and 0.1 % SDS) (Millipore). Membranes were blocked in 5% (wt/vol) dried milk in tris-buffered saline (TBS) and Tween-20 (TBST) for 1 h at room temperature. First, blots were blocked for an hour in TBST (TBS with 0.5% Tween-20) with 5% nonfat skimmed milk. Then, primary antibodies MMP9 (ab228402), ICAM-1 (ab222736), CHI3L1 (ab93034) from Abcam, and VCAM-1 (32653) from Cell Signaling Technology with (1:1,000) were used to probe them overnight at 4°C. After the membranes were washed three times with TBST, horseradish peroxidase-conjugated secondary antibodies from Bio-Rad were added and left to react for 1 h at room temperature. Protein bands were visualized using the Super-Signal West-Femto Chemiluminescent Substrate (Thermo Scientific, Rockford, IL). Membranes were treated with the substrate (Super Signal West Femto) from Thermo Scientific to reveal the protein bands acquired on Invitrogen i-Bright Imaging Systems, and band intensities were quantified for semiquantitative analysis using ImageJ software.

### Quantification of IL-33 in human vitreous

All endophthalmitis cases underwent complete ophthalmic examinations, which included B-scans, slit-lamp biomicroscopy, and visual acuity measurements at the time of diagnosis, and microbiology lab testing reported that all 60 cases were culture-positive for bacterial or fungal etiology. The median duration from symptom onset to vitreous sampling for bacterial endophthalmitis was 48 h (range: 24–72 h), and for fungal endophthalmitis, it was 96 h (range: 72–168 h). Total protein concentration in vitreous samples was determined using the BCA assay, and each sample was standardized to a concentration of 300 µg/mL. Cytokine estimation was performed using the MILLIPLEX Human Cytokine/Chemokine Magnetic Bead Panel (Merck, catalog number: LXSAHM-05), following the manufacturer’s instructions. Briefly, 25 µL of vitreous samples were loaded onto a 96-well plate provided in the kit and incubated with antibody-coated magnetic beads. After incubation, detection antibodies and streptavidin-phycoerythrin were added, followed by a final incubation step. The plates were then gently washed with wash buffer, and the beads were resuspended in sheath fluid. Cytokine levels were measured and analyzed using the Luminex MAGPIX Multiplex System (Merck Millipore).

### Cell cytotoxicity assay

Cell death was quantified by measuring LDH release into the supernatant of cultured mouse BMDMs at various time points during bacterial and fungal infection, as specified in the assay protocol. This was performed using the Pierce LDH Cytotoxicity Assay Kit (Catalog No. 88954), following the manufacturer’s protocol. Additionally, cell death was further evaluated using the SYTOX Green Nucleic Acid Stain (Thermo Fisher, Catalog No. S7020), which selectively labels cells with compromised membranes. The concurrent use of the SYTOX assay served to validate the LDH assay results, providing an additional measure of cell membrane integrity and cytotoxicity under infection conditions.

### Statistical analysis

Data are expressed as the mean ± SD. Statistical significance among experimental groups was determined using the student’s *t*-test or one-way analysis of variance (ANOVA) with Tukey’s post hoc test, with a *P* value of <0.^05^ considered significant. All analyses were performed using GraphPad Prism ^10^ (GraphPad Software, La Jolla, CA, USA).

## SUPPLEMENTAL MATERIAL

Supplemental material iai.00183-25-s0001.pdf Fig. S1 to S4; Tables S1 and S2.

ASM does not own the copyrights to Supplemental Material that may be linked to, or accessed through, an article. The authors have granted ASM a non-exclusive, world-wide license to publish the Supplemental Material files. Please contact the corresponding author directly for reuse.
