# Supplementary material for: Proteomic profiling reveals immunomodulatory role of IL-33 in ocular bacterial and fungal infections
Source: Infect Immun. 2025 Jun 13;93(7):e00183-25. doi: 10.1128/iai.00183-25 (PMC12234442; doi:10.1128/iai.00183-25)
Supplement: Supplemental material — Fig. S1 to S4; Tables S1 and S2. [file iai.00183-25-s0001.pdf]

Supplemental data

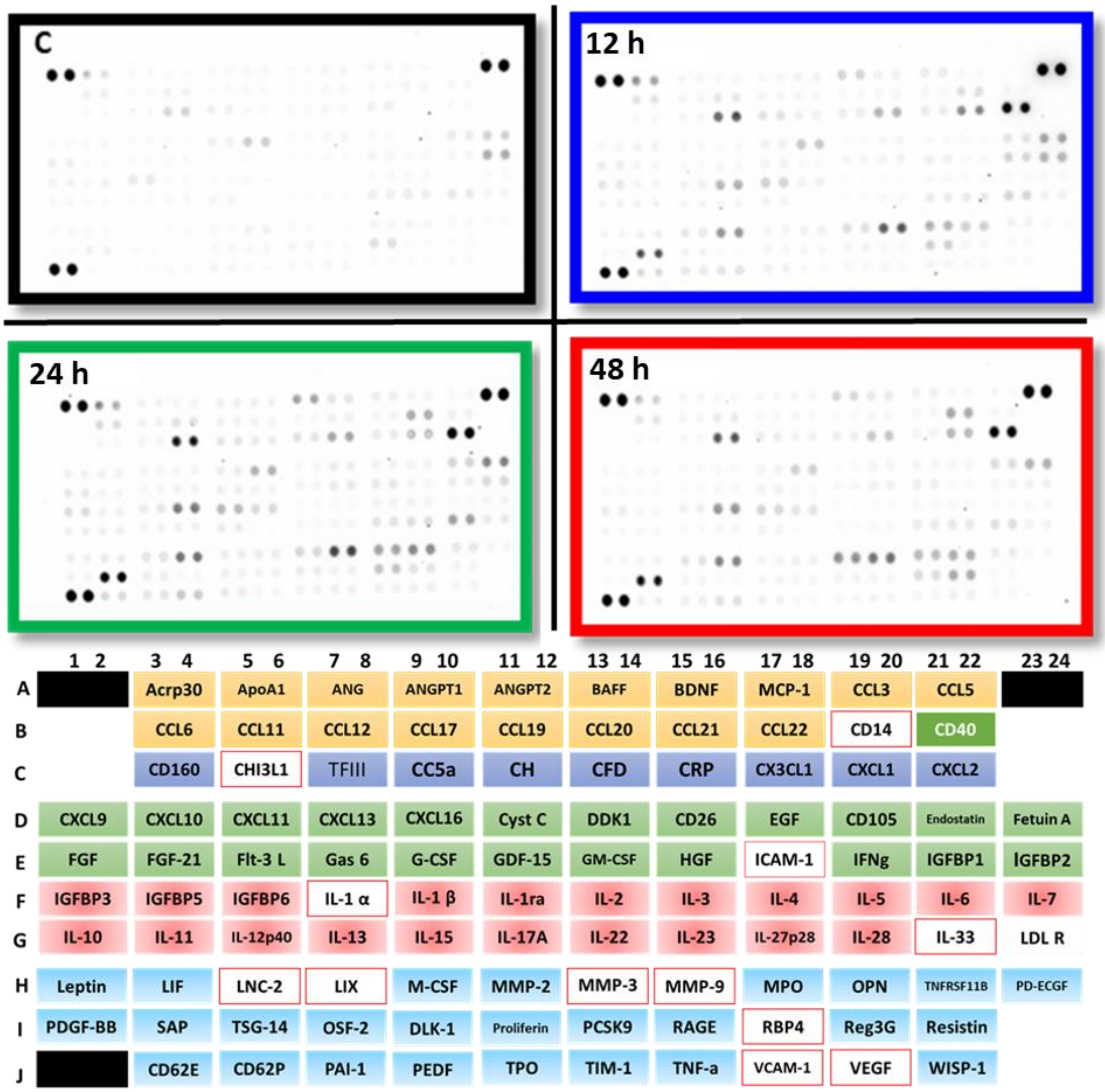

Fig. S1. Proteome profiler cytokine array from whole eye lysate during *S. aureus* endophthalmitis. Raw cytokine dot-blot images showing different time points: Control (C), 12, 24, and 48-hours *S. aureus* post-infection. Each dot represents a cytokine intensity, with darker spots indicating higher expression. A reference map below indicates the identity and location of each cytokine on the membrane.

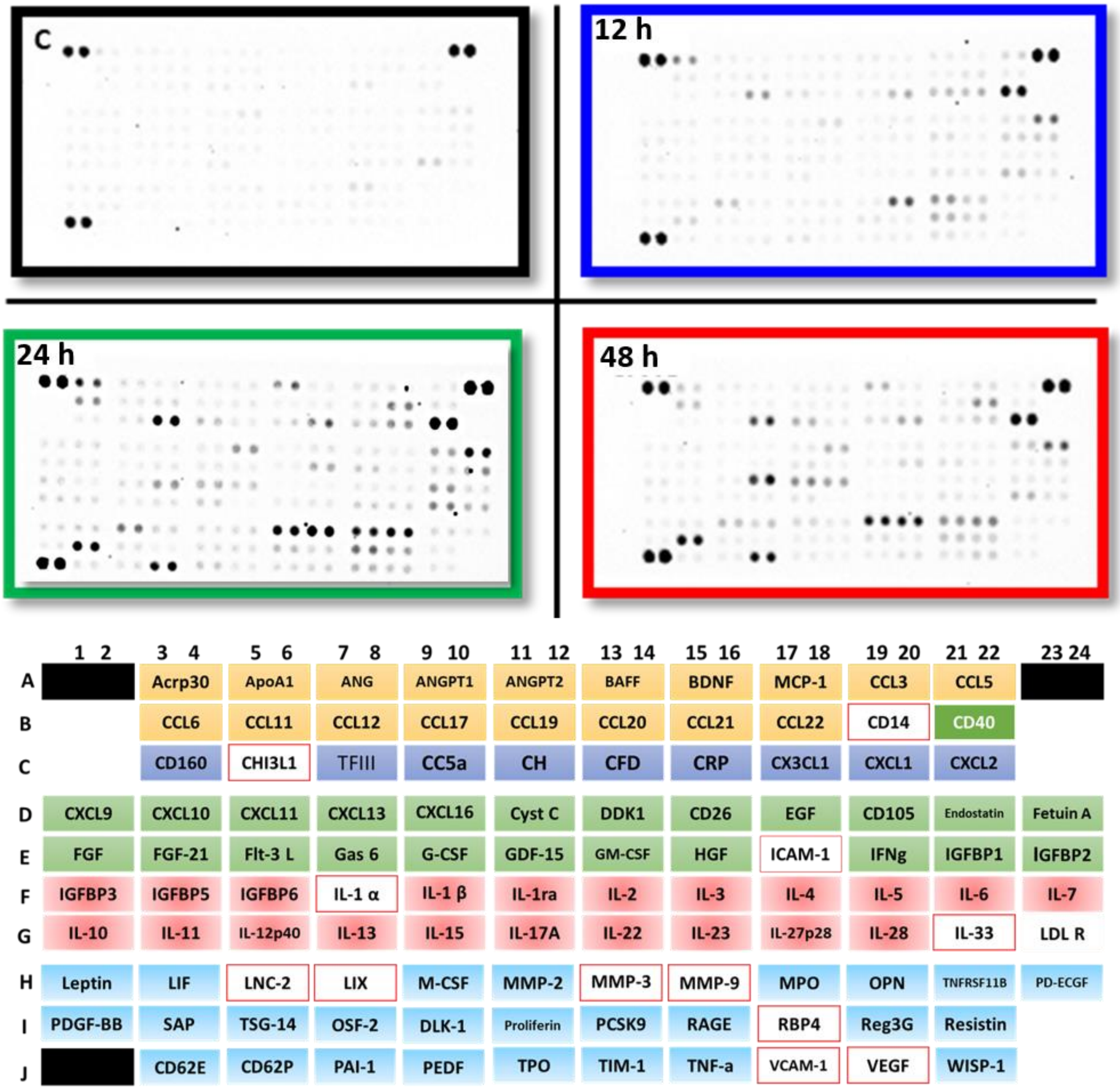

Fig. S2. Proteome profiler cytokine array from retinal lysate during *S. aureus* endophthalmitis. Raw cytokine dot-blot images showing different time points: Control (C), 12, 24, and 48-hours *S. aureus* post-infection. Each dot represents a cytokine intensity, with darker spots indicating higher expression. A reference map below indicates the identity and location of each cytokine on the membrane.

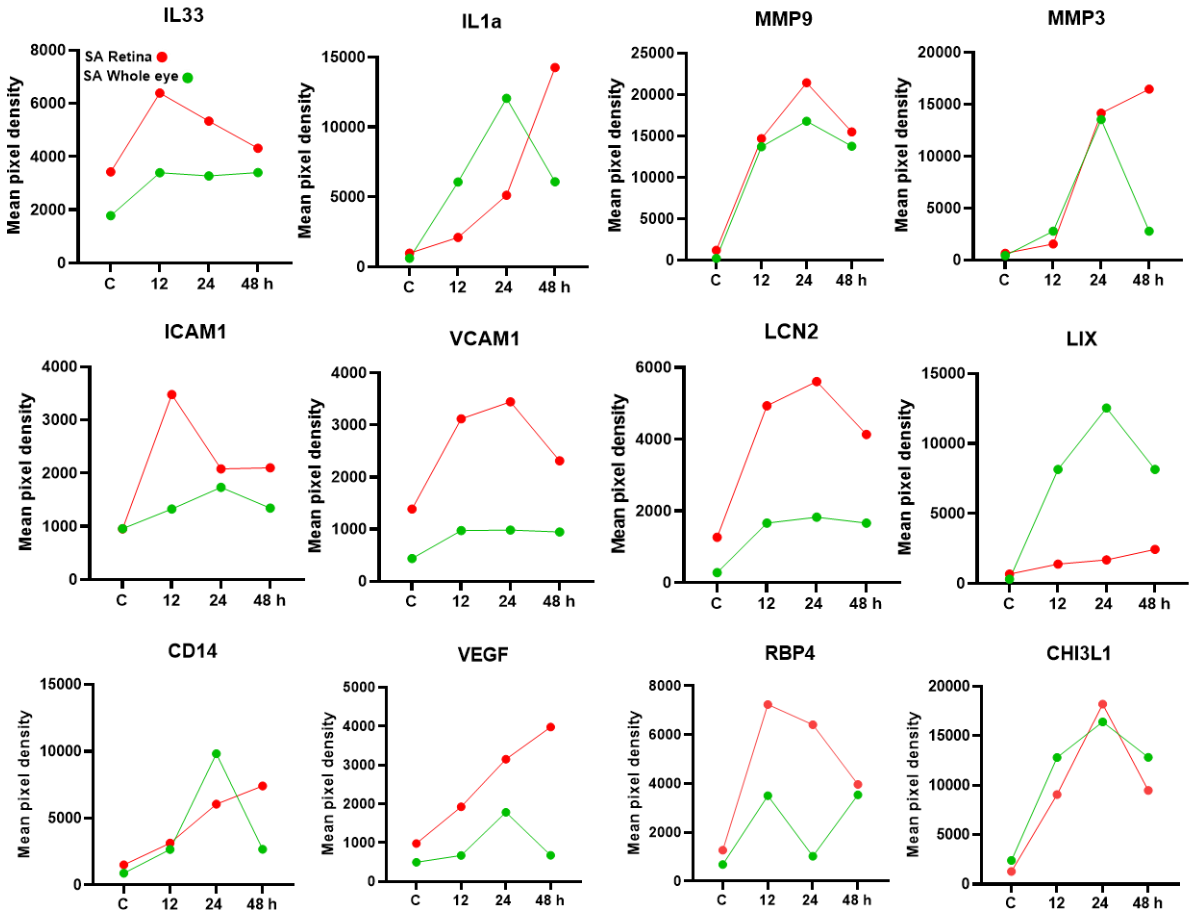

Fig. S3: Comparative analysis of proteins in the whole eye lysate versus retinal lysate during *S. aureus* endophthalmitis. The protein profiles of the retina (Red line) and whole eye (green line) infected *S. aureus* endophthalmitis at 12, 24, and 48-hours post-infection were compared heat-to-head to visualize the relative expression. The data represented are the culmination of two independent experiments and are shown as mean.

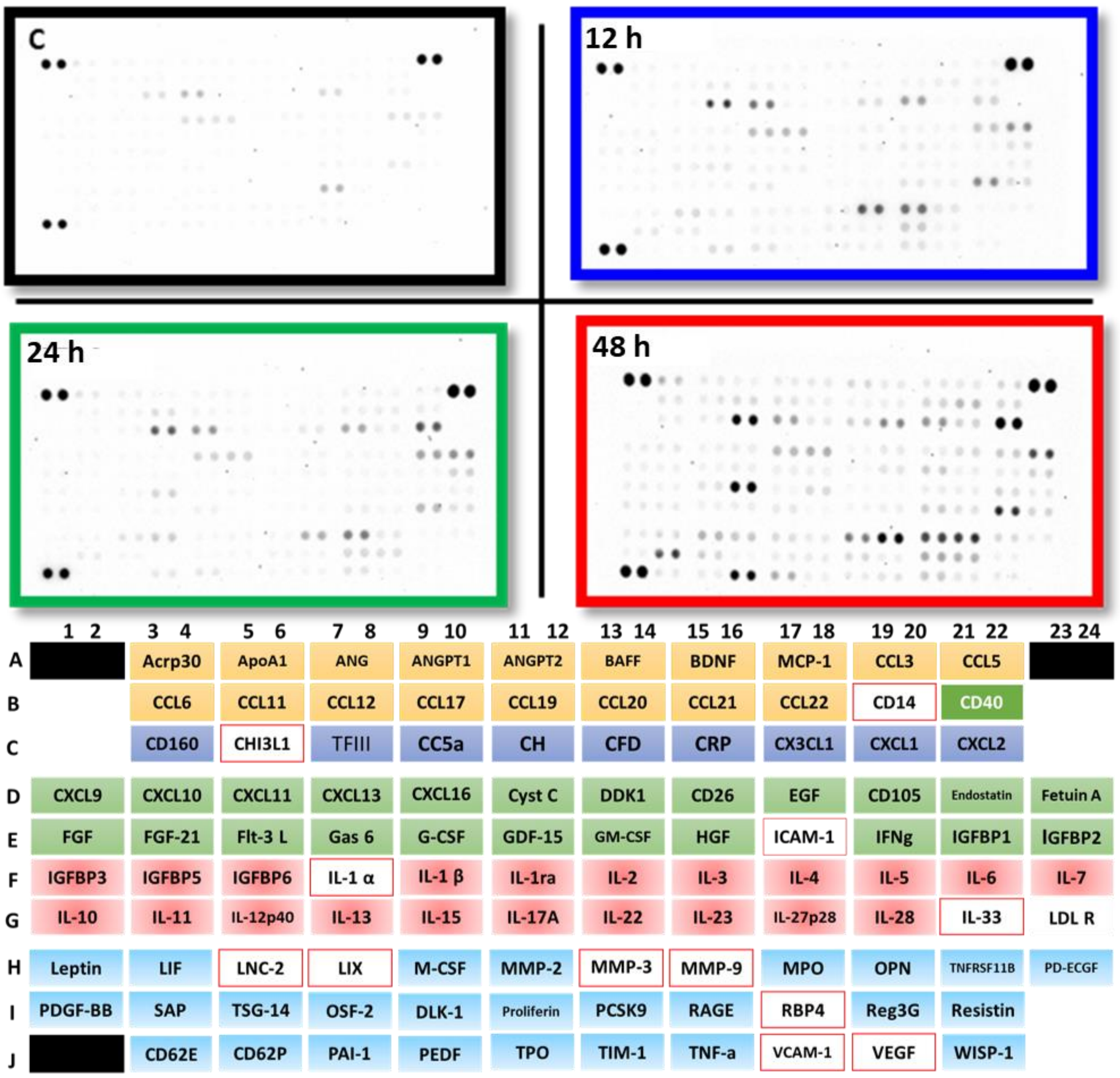

Fig. S4: Proteome profiler cytokine array from retinal lysate during *A. fumigatus* endophthalmitis. Raw cytokine blot images showing different time points: Control (C), 12, 24, and 48-hours *A. fumigatus* post-infection. Each dot represents a cytokine intensity, with darker spots indicating higher expression. A reference map below indicates the identity and location of each cytokine on the membrane.

Supplementary Table 1. List of molecules exhibiting significant change across all experimental groups

SA WHOLE EYE	SA RETINA	AF RETINA
BAFF/BLyS/TNFSF13B	BAFF/BLyS/TNFSF13B	CCL2/JE/MCP-1
CCL2/JE/MCP-1	C1q R1/CD93	CCL3/CCL4 MIP-1 α/β
CCL6/C10	CCL3/CCL4 MIP-1 α/β	CCL5/RANTES
CCL12/MCP-5	CCL5/RANTES	CCL12/MCP-5
CD14	CCL6/C10	CCL17/TARC
Chitinase 3-like 1	CD14	CCL21/6Ckine
Coagulation Factor III	Chitinase 3-like 1	CCL22/MDC
Complement Component C5/C5a	Complement Factor D	CD14
Complement Factor D	C-Reactive Protein/CRP	CD40/TNFRSF5
C-Reactive Protein/CRP	CXCL1/KC	Chitinase 3-like 1
CXCL1/KC	CXCL2/MIP-2	Coagulation Factor III
CXCL2/MIP-2	Cystatin C	Complement Factor D
Endostatin	Endostatin	C-Reactive Protein/CRP
Fetuin A/AHSG	Fetuin A/AHSG	CX3CL1/Fractalkine
G-CSF	HGF	CXCL1/KC
GM-CSF	ICAM-1/CD54	CXCL2/MIP-2
HGF	IL-1 alpha/IL-1F1	CXCL10/IP-10
IGFBP-1	IL-6	CXCL16
IGFBP-3	Lipocalin-2/NGAL	Cystatin C
IL-1 alpha/IL-1F1	LIX	Endoglin/CD105
IL-1 beta/ IL-1F2	MMP-3	Endostatin
IL-1ra/IL-1F3	MMP-9	Fetuin A/AHSG
IL-3	Myeloperoxidase	Flt-3 Ligand
IL-6	Osteopontin OPN	Gas 6
IL-33	Osteoprotegerin	GDF-15
Lipocalin-2/NGAL	PD-ECGF	GM-CSF
LIX	Pentraxin 2/SAP	HGF
MMP-3	Pentraxin 3/ TSG-14	ICAM-1/CD54
MMP-9	Periostin/OSF-2	IFN-gamma
Myeloperoxidase	RAGE	IGFBP-1
Osteopontin (OPN)	RBP4	IGFBP-2
Osteoprotegerin	Reg3G	IGFBP-6
Pentraxin 2/SAP	Resistin	IL-1 alpha/IL-1F1
Pentraxin 3/ TSG-14	E-Selectin/CD62E	IL-1ra/IL-1F3
IPeriostin/OSF-2	P-Selectin/CD62P	IL-3
RAGE	Serpin F1/PEDF	IL-5
Reg3G	Thrombopoietin	IL-6

E-Selectin/CD62E	TIM-1/KIM-1/ HAVCR	IL-7
P-Selectin/CD62P	TNF-alpha	IL-15
Serpin E1/PAI-1	VCAM-1/CD106	IL-22
VCAM-1/CD106	VEGF	IL-23
	WISP-1/CCN4	IL-27p28
		IL-28
		IL-33
		LDL R
		Lipocalin-2/NGAL
		LIX
		M-CSF
		MMP-2
		MMP-3
		MMP-9
		Myeloperoxidase
		Osteopontin OPN
		Osteoprotegerin
		PD-ECGF/ Thymidine phosphorylase
		Pentraxin 2/SAP
		Pentraxin 3/ TSG-14
		Proprotein Convertase 9/ PCSK9
		RAGE
		RBP4
		Reg3G
		Resistin
		Serpin E1/PAI-1
		Serpin F1/PEDF
		TIM-1/KIM-1/ HAVCR
		VCAM-1/CD106
		VEGF
		WISP-1/CCN4

Supplementary Table 2: Demographic details of the study group

Category	Gram-Positive (n=20)	Gram-Negative (n=20)	Fungal (n=20)	Control (n=20)
**Mean Age (years) ± SD (Range)**	30.7 ± 23.9 (2-72)	45.3 ± 23.8 (4-74)	44.8 ± 18.5 (2m-68)	54.4 ± 14.8 (19-72)
**Male (%)**	70% (n=14)	70.0% (n=14)	70% (n=14)	75% (n=15)
**Female (%)**	30% (n=6)	30.0% (n=6)	30% (n=6)	25% (n=5)
**Clinical Presentation**				
**Traumatic Endophthalmitis**	50% (n=10)	30% (n=6)	15% (n=3)	-
**Endogenous Endophthalmitis**	25% (n=5)	45% (n=9)	55% (n=11)	-
**Post-operative Endophthalmitis**	25% (n=5)	20% (n=4)	20% (n=4)	-
**Post-traumatic Panophthalmitis**	-	5% (n=1)	-	-
**Traumatic Cataract with Endophthalmitis**	-	-	10% (n=2)	-
**Retinal Detachment**	-	-	-	65% (n=14)
**Vitreous Haemorrhage**	-	-	-	10% (n=2)
**Macular Hole**	-	-	-	10% (n=2)
**Membrane Peeling**	-	-	-	10% (n=2)
**Initial Visual Acuity**				
**Perception of Light (PL) Projection of Rays (PR)**	50% (n=10)	50% (n=10)	50% (n=10)	20% (n=4)
**Hand Motion (HM+)**	25% (n=5)	25% (n=5)	30% (n=6)	30% (n=6)
**Counting Fingers (CF) or Better**	25% (n=5)	25% (n=5)	20% (n=4)	50% (n=10)
**Final Visual Acuity**				
**PL+ PR or Worse**	33.3% (n=7)	30% (n=6)	35% (n=7)	5% (n=1)
**Hand Motion (HM+)**	23.8% (n=5)	25% (n=5)	30% (n=6)	15% (n=3)
**Counting Fingers (CF) or Better**	19% (n=4)	30% (n=6)	25% (n=5)	65% (n=13)
**Deferred**	23.8% (n=4)	15% (n=3)	10% (n=2)	5% (n=1)
